# Editorial: COVID-19: From bedside to follow-up

**DOI:** 10.3389/fmed.2023.1155049

**Published:** 2023-02-22

**Authors:** Jesper Damsgaard Gunst, Sara Cajander

**Affiliations:** ^1^Department of Infectious Diseases, Aarhus University Hospital, Aarhus, Denmark; ^2^Department of Clinical Medicine, Aarhus University, Aarhus, Denmark; ^3^Department of Infectious Diseases, Faculty of Medicine and Health, Örebro University, Örebro, Sweden

**Keywords:** COVID-19, SARS-CoV-2, bedside, follow-up, immunogenicity, post-acute COVID-19 syndrome, post-acute COVID-19 condition

Many Research Topics have been established regarding coronavirus disease 2019 (COVID-19) “*From Bench to Bedside*,” i.e., the research process by which laboratory results generated at the bench are directly used at the bedside to treat the patients. In this Research Topic entitled “*COVID-19: Bedside to Follow-up*,” we explore the effects of severe acute respiratory syndrome coronavirus 2 (SARS-CoV-2) infection on the clinical course from bedside to follow-up, as well as the effects of SARS-CoV-2 vaccination over time.

In comparison studies, perfect controls do not differ genetically from the cases, as seen with monozygotic twin pairs. In a case report, de Castro et al. present follow-up data from a monozygotic twin pair after severe COVID-19 before vaccination. These twin brothers were both admitted to the intensive care unit but one of them, who had a longer hospitalization, had persistent muscle weakness and fatigue during long-term follow-up. Consequently, this report highlights the role of non-genetic factors in the pathogenesis of severe COVID-19 and post-COVID-19 conditions. Data from large databases, before the introduction of COVID-19 vaccines, have shown that the most relevant non-genetic risk factor is age (1). The age-related risk of severe COVID-19 increases gradually and is more than doubled in the age interval 60–69 years compared with 50–59 years. Other relevant risk factors for disease progression among the unvaccinated include male sex, obesity (BMI > 35), and co-morbidities, such as cancer, organ transplantation, and chronic kidney, heart, and lung disease. Today, in the Omicron era, advanced age (≥65 years) is still one of the most relevant risk factors for developing severe COVID-19 and death, followed by vaccination status and immunocompromised conditions (2–4). Although the risk of severe COVID-19 or death for unvaccinated patients with Omicron is lower than with Delta variants, the risk is still relevant and similar to ancestral lineages (5).

For prediction models estimating the mortality risk in acute COVID-19 to be useful, they need to be based on easily accessible clinical parameters and routinely available laboratory tests. By using point-based scores of only four parameters, age, oxygen saturation, C-reactive protein, and creatinine levels, in a large cohort in Austria, Horvath et al. validated their mortality risk prediction tool (6), which can be assessed at (https://www.cbmed.at/covid-19-risk-calculator/). Robertson et al. present data from 19 moderate and 104 severe COVID-19 patients compared with 20 matched disease-free controls on another biomarker, the level of the circulating soluble form of angiotensin-converting enzyme 2 (sACE2) protein. The level of sACE2 decreased with disease severity in men but increased with disease severity in women, suggesting sex-specific differences in how the level of sACE2 correlates with COVID-19 severity. A longitudinal study has shown that sACE2 levels are lower in children than in adults and increases only in males from the age of 12 years (7). Why opposite trends in sex-specific sACE2 levels are observed among severe COVID-19 patients needs to be further investigated.

In a substudy of the Danish National Cohort Study of Effectiveness and Safety of SARS-CoV-2 vaccines (ENFORCE) cohort (8), Hvidt et al. present direct comparative analyses of four COVID-19 vaccines following primary and booster vaccination, focusing on the vaccine-induced humoral immune responses against diverse SARS-CoV-2 variants. The COVID-19 vaccine immunogenicity as measured by SARS-CoV-2 spike IgG levels and antibody neutralization titers reached similar levels among the four COVID-19 vaccines.

Early in the pandemic, there was insufficient knowledge about the risk of developing severe COVID-19 among immunocompromised patients. Today, we know that organ transplant recipients are poor vaccine responders and are one of the most important risk group for developing severe COVID-19, as presented in the umbrella review on kidney health by Yang et al. Interestingly, Benning et al., in a prospective observational cohort study, show that COVID-19 vaccine immunogenicity could be improved in kidney transplant recipients with a fourth vaccine dose after short-term withdrawal of the immunosuppressant mycophenolic acid. However, for safety reasons, short-term withdrawal of mycophenolic acid can only be considered in kidney transplant recipients without prior or current anti-HLA donor-specific antibodies.

To prevent severe COVID-19, Ouyang et al. theoretically reviewed the pros and cons of SARS-CoV-2 pre-exposure prophylaxis using antivirals, as well as other anti-SARS-CoV-2 agents, for high-risk groups, including healthcare workers, immunodeficient individuals, and poor vaccine responders. A personalized medicine approach consisting of risk stratification and decisions on early antiviral treatments based on measurements of an individual's vaccine response is an attractive option. However, this is often hindered by limitations in screening resources. An example on how this can be facilitated is shown in the study by Schmetzer et al., in which healthcare workers and patients with immune-mediated inflammatory diseases, who are likely to be poor vaccine responders, used self-collection of capillary blood and saliva to determine COVID-19 vaccine immunogenicity. Despite a limited study cohort size (*n* = 60), self-sampling was shown to be accurate and feasible. The study was conducted under controlled conditions but self-collection could potentially also be used “at home” to increase flexibility. Follow-up studies from larger cohorts are needed to conclude the effectiveness of self-collection to determine COVID-19 vaccine immunogenicity in clinical practice.

Health outcomes after 15 months of follow-up are described by Sun et al. in a cohort of 534 COVID-19 patients hospitalized in Wuhan during the first wave. The most prevalent self-reported symptoms were sleep disorders (19%) and fatigue (17%). Of note, there are generally good correlations between self-reported symptoms and validated health scores (9). In multivariate logistic regression analyses, the risk of sleep disorders was significantly associated with females compared with males, and glucocorticoid use during hospitalization was significantly associated with an increased risk of fatigue. Five percent of the COVID-19 patients suffered from post-traumatic stress disorder (PTSD), which was significantly associated with persistent symptoms during follow-up compared with no persistent symptoms in a multivariate logistic regression analysis. No significant associations were observed between COVID-19 severity and sleep disorders, fatigue, or PTSD. Of note, even subtle cognitive symptoms have previously been shown to worsen when returning to normal life following viral infection (10). Reports on the Omicron variant from East Asia are sparse, but Shen et al. present clinical characteristics and 1-month recovery of subjective hyposmia in a cohort of 349 hospitalized patients infected with Omicron. Among these non-severe COVID-19 patients, the prevalence of Omicron-related hyposmia was 6%. The patients with hyposmia had more clinical symptoms than patients without hyposmia, which might have contributed to longer hospitalization.

We look forward to the many reports on data from bedside to long-term follow-up after COVID-19, as well as the effects of SARS-CoV-2 booster vaccination over time in the coming years. Lastly, we would like to thank all the authors and reviewers for their contribution.

## Author contributions

JDG drafted the manuscript. All authors critically revised the manuscript for important intellectual content. All authors contributed to the article and approved the submitted version.
